# Effect of Age and Embryo Morphology on Live Birth Rate After Transfer of Unbiopsied Blastocysts

**DOI:** 10.5935/1518-0557.20200101

**Published:** 2021

**Authors:** Michael Awadalla, Ashley Kim, Nicole Vestal, Jacqueline Ho, Kristin Bendikson

**Affiliations:** 1 Division of Reproductive Endocrinology and Infertility, Department of Obstetrics and Gynecology, Keck School of Medicine, University of Southern California, Los Angeles, California; 2 Department of Obstetrics and Gynecology, Kaiser Permanente, Los Angeles, California; 3 Keck School of Medicine, University of Southern California, Los Angeles, California

**Keywords:** *in vitro* fertilization, mathematical modeling, blastocyst, embryo transfer, live birth rate

## Abstract

**Objective:**

To determine the rate of live birth per blastocyst based on morphology and oocyte age using data from a single center.

**Methods:**

This is a mathematical analysis and model building study of autologous blastocyst stage embryo transfers at a University-affiliated center. A total of 448 blastocyst stage embryos were transferred in 244 fresh and frozen embryo transfers from May 2015 through April 2018. Blastocyst morphology was divided into good, fair, and poor overall morphology grades. Each embryo transfer was modeled as an equation equating the sum of the unknown live birth rates of the transferred embryos to the number of live births that resulted. The least squares solution to the system of embryo transfer equations was determined using linear algebra.

**Results:**

Trophectoderm morphology was a better predictor of live birth rate than inner cell mass morphology. Embryos graded AA/AB/BA (good) had the highest live birth rates followed by BB/CB (fair), and BC/CC (poor). In our youngest age group (25-32 years) live birth rates per embryo were 51% for good, 39% for fair, and 25% for poor quality embryos. In our oldest age group (40-44 years) the live birth rates per embryo were 22% for good, 14% for fair, and 8% for poor quality embryos.

**Conclusions:**

These techniques can help analyze small datasets such as those from individual clinics to aid in determining the ideal number of embryos to transfer to achieve live birth while limiting the risk of multiple gestations.

## INTRODUCTION

Current guidance on the limits to the number of embryos to transfer at one time is based on expert opinion. Individual clinics are encouraged to use their own data to determine a safe number of embryos to transfer to limit the risk of multiple gestations (Penzias *et al*., 2017). Despite these recommendations, there is limited information on how to develop a quantitative model for predicting transfer outcomes and even less information on how to do this with a small dataset such as that from an individual clinic. Determining the live birth rate per embryo is an essential starting point for predicting transfer outcomes.

There are four main difficulties with using models to determine an appropriate number of embryos to transfer. First, analyzing transfers of multiple embryos poses challenges and most clinics do not have sufficient data using only single unbiopsied embryo transfers. Other studies have analyzed single embryo transfers from multiple clinics using data from the Society for Assisted Reproductive Technology Clinic Outcomes Reporting System (SART CORS) database (Luke *et al*., 2014; Thompson *et al*., 2013). However, the current SART embryo grading system does not specify how overall embryo grades of good/fair/poor are to be assigned (Racowsky *et al.*, 2010). This assignment remains subjective and lack of standardization between clinics limits studies that pool data from multiple clinics. Second, it is challenging to incorporate multiple embryo characteristics into a single model. Most studies analyze combinations of blastocyst expansion stage, inner cell mass (ICM) grade, trophectoderm (TE) grade, overall grade, oocyte age at retrieval, fresh or frozen transfer, and other factors. Since there is little uniformity across studies, comparison of results is limited. Third, dealing with age-related fertility decline is difficult. Fixed age groups of less than 35 years, 35-37 years, 38-40 years, 41-42 years, and greater than 42 years are commonly used. This makes it difficult to apply study results to patients at the extremes of the age groups such as patients 37 or 38 years old (Penzias *et al*., 2017; Thompson *et al*., 2013). Lastly, since many additional factors affect all embryos transferred concurrently (uterine, culture, transfer, environmental, and others), multiple gestations result more frequently than would be expected if each embryo implanted independently of the others (Torsky *et al*., 2005).

The ultimate goal of in vitro fertilization (IVF) is to maximize clinical pregnancy and live birth rates (LBRs) while limiting the risk of multiples (Penzias et al., 2017; Pfeifer *et al*., 2012a). Multiple gestation is associated with increased rates of preterm labor and increased maternal and fetal morbidity and mortality including low birth rate, neonatal intensive care unit admission, caesarean delivery and gestational diabetes (Pfeifer *et al*., 2012b). Compared to single blastocyst transfer, double blastocyst transfer increases the pregnancy rate but is associated with an increase in twin births from 0-2% to 16-33% (Berin *et al*., 2011; Devine *et al*., 2015; Yanaihara *et al*., 2008). Although blastocyst transfer is associated with higher live birth rates per embryo than cleavage stage transfer, transfer of multiple blastocysts is often needed to maintain pregnancy rates in poor prognosis patients (Penzias *et al*., 2018; 2017). It is essential to develop a more precise and quantitative model to guide the number of embryos that should be transferred at one time.

The first objective of this study is to determine how to best group specific blastocyst stage embryo morphologies into good/fair/poor overall morphology grades. The second objective is to determine the rate of live birth per blastocyst based on overall morphology grade and oocyte age using data from a single center.

## MATERIALS AND METHODS

### Study Population

**Figure 1:**
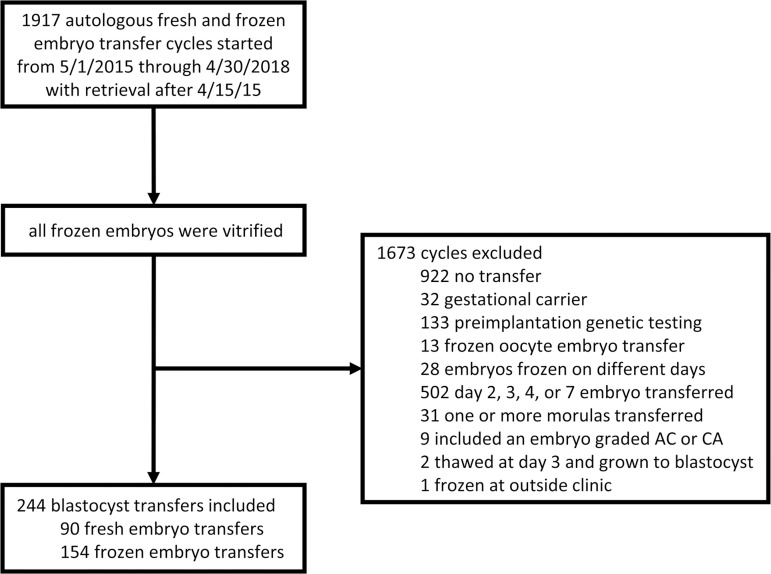
Flow diagram with inclusion and exclusion criteria.

A model building analysis was performed using data from 173 patients during 244 fresh and frozen autologous blastocyst embryo transfers. Cycles were included if the cycle was started from May 2015 through April 2018 and oocyte retrieval occurred after April 15^th^ 2015. The following exclusion criteria were used: no transfer, gestational carrier used, preimplantation genetic testing, frozen oocyte embryo transfer, embryos frozen on different days, day 2/3/4/7 embryo transferred, one or more morulas transferred, transfer of an embryo graded AC or CA (since these grades are rarely assigned), embryo thawed at day 3 and grown to blastocyst, and embryos frozen at an outside clinic (Figure 1). Demographics and transfer cycle characteristics are included in Table 1. A total of 448 embryos were transferred for an average of 1.8 embryos transferred at once. These embryo transfers resulted in a total of 175 sustained implantations (defined as a fetal heartbeat at 6 to 8 weeks gestation) and 147 live born infants (89 singleton deliveries and 29 twin deliveries).

**Table 1:** Demographics and transfer cycle characteristics

	Mean (SD)
Age (years)	35.1 (4.2)
BMI	25.7 (6.2)
Race/Ethnicity White Asian Hispanic African American Multiple Unknown	34.0% 28.3% 23.4% 6.2% 5.7% 2.5%
Transfer type n (%) fresh blastocyst frozen blastocyst	90/244 (36.9%) 154/244 (63.1%)
Number of embryos transferred n (%) 1 2 3 4	66 (27.0%) 154 (63.1%) 22 (9.0%) 2 (0.8%)
Day of transfer n (%) Day 5 Day 6	233/244 (95.5%) 11/244 (4.5%)

### Blastocyst Grading

We used a modified version of the grading system described by Gardner & Schoolcraft (1999). Each blastocyst was given a score for degree of expansion from 1 (early blast) to 6 (hatched blast) followed by a two-letter grade with the first letter indicating the ICM grade, and the second letter indicating the TE grade. The ICM grade was assigned as follows: A for tightly packed with many cells, B for loosely grouped with several cells, and C for very few cells. The TE grade was assigned as follows: A for many cells forming a cohesive epithelium, B for few cells forming a loose epithelium, and C for very few cells. For frozen embryo transfers the embryo grade prior to vitrification was used. Since it was rare for the ICM and TE scores to differ by more than one letter grade, transfers including embryos graded AC or CA were excluded from this analysis because there was not enough data to make inferences about these embryos. The distribution of embryo morphology is included in Table 2. For standardization, all embryologists at our center were trained in embryo grading by the same laboratory director. Details on IVF protocols used are included in the supplemental methods section.

**Table 2:** Embryo morphology distribution

Morphology	Percent
AA	10.3%
AB	8.3%
BA	4.5%
BB	49.6%
CB	3.8%
BC	6.9%
CC	16.7%
**Overall Morphology Grade**	**Percent**
Good (AA/AB/BA)	23.0%
Fair (BB/CB)	53.3%
Poor (BC/CC)	23.7%

### Determination of Live Birth Rates for Each Embryo Morphology and Overall Morphology Grade

Each of the 244 embryo transfers was modeled as an equation with seven unknown variables with each variable representing the live birth rate (LBR) for an embryo with a given morphology (AA, AB, BA, BB, BC, CB, and CC). The coefficients (N) in the equation represent the number of embryos of each morphology that were transferred. The sum of the coefficients multiplied by their respective unknown variables was set equal to the number of live births that resulted from the embryo transfer (Equation 1). The equations were solved for the unknown variables with linear algebra to give the least squares solution to the system of equations using MATLAB version 9.5 (MathWorks). This analysis was performed for all embryo transfers and separately for all transfers in subjects less than 38 years old to control for age (wle 3). The seven embryo morphologies were then grouped into good (AA/AB/BA), fair (BB/CB), and poor (BC/CC) categories of overall morphology grade based on this analysis. Live birth rates for embryos in each category of overall morphology grade were determined by repeating the analysis for embryo transfers grouped into different age groups based on age at oocyte retrieval (Equation 2 and Table 4).

**Table 3:** Best fit live birth rate per blastocyst for each of seven embryo morphologies

	Morphology						
	AA	AB	BA	BB	CB	BC	CC
overall grade	good	good	good	fair	fair	poor	poor
all ages combined LBR per embryo n (embryos) average age	54% 46 34	44% 37 34	55% 20 33	29% 222 36	32% 17 35	15% 31 36	15% 75 38
< 38 years LBR per embryo n (embryos) average age	58% 35 33	56% 29 33	53% 18 32	34% 143 33	46% 12 34	27% 17 33	33% 29 33

These results were used to assign overall morphology grades of good, fair, and poor.

**Table 4:** Best fit live birth rate per blastocyst by overall morphology grade stratified by age

	Overall Grade		
	Good	Fair	Poor
Morphology	AA/AB/BA	BB/CB	BC/CC
all ages combined LBR per embryo n (embryos) average age	51% 103 34	30% 239 36	15% 106 37
< 38 years LBR per embryo n (embryos) average age	56% 82 33	35% 155 33	31% 46 33
≥ 38 years LBR per embryo n (embryos) average age	30% 21 39	22% 84 40	8% 60 40
< 35 years LBR per embryo n (embryos) average age	54% 55 31	34% 95 32	24% 22 31
35-37 years LBR per embryo n (embryos) average age	60% 27 36	37% 60 36	37% 24 36
38-40 years LBR per embryo n (embryos) average age	35% 17 39	28% 49 39	12% 2 39

Equation 1:

$$\begin{array}{c} N_{\mathrm{AA}}*{\mathrm{LBR}}_{\mathrm{AA}}+N_{\mathrm{AB}}*{\mathrm{LBR}}_{\mathrm{BA}}+N_{\mathrm{BB}}*{\mathrm{LBR}}_{\mathrm{BB}}+\\ N_{\mathrm{BC}}*{\mathrm{LBR}}_{\mathrm{BC}}+N_{\mathrm{CB}}*{\mathrm{LBR}}_{\mathrm{CB}}+N_{\mathrm{CC}}*{\mathrm{LBR}}_{\mathrm{CC}}=\mathrm{number}\;\mathrm{of}\;\mathrm{live}\;\mathrm{births} \end{array}$$

Equation 2:

$$N_{\mathrm{good}}*{\mathrm{LBR}}_{\mathrm{good}}+N_{\mathrm{fair}}*{\mathrm{LBR}}_{\mathrm{fair}}+N_{\mathrm{poor}}*{\mathrm{LBR}}_{\mathrm{poor}}=\mathrm{number}\;\mathrm{of}\;\mathrm{live}\;\mathrm{births}$$

### Moving Centered Age Groups

Live birth rates for each of the three groups of overall morphology grade (good, fair, and poor) were determined by age at oocyte retrieval in 1-year increments for patients aged 33 to 39 years old (with age rounded to the nearest integer). For each age we determined live birth rates using embryo transfers of patients who were three years younger to three years older. This was done to create a 7-year age group centered on the age of interest. For example, data from patients aged 30 to 36 years old at the time of oocyte retrieval was used to determine the live birth rates for patients aged 33 years old. Data from patients aged 31 to 37 years old was used to determine the live birth rates for patients aged 34 years old. We performed the entire analysis with 1-year, 3-year, 5-year, 7-year, and 9-year age groups. The 7-year age group was chosen because it was the smallest age group that visually smoothed out random variation in the data. Since the average age in each 7-year age group differed slightly from the original center age, linear interpolation was used to re-center the live birth rates at the intended center age. Two additional age groups were used for patients younger than 33 years or older than 39 years.

### Statistical Analysis and Confidence Interval Determination

A priori power analysis is not possible with this type of analysis since the result depends on the numbers and types of embryos transferred and the live birth rates. Thus, this is a data limited study. Computer simulation in MATLAB was used to perform bootstrapping to determine the confidence intervals for the live birth rates since there are no existing statistical methods applicable to this type of modeling. This methodology is detailed further in the supplemental methods. Stratification by age was used to control for the confounding effect of age on the relationship between morphology and live birth rates. For each 7-year moving age group (and the less than 33-year and older than 39-year age groups) a separate analysis was performed to determine the 95% confidence intervals for the live birth rates in that group (Table 5).

**Table 5:** Live birth rates with 95% confidence intervals by age and overall morphology grade

Age Group (actual average age)	Good (AA/AB/BA)	Fair (BB/CB)	Poor (BC/CC)
25-32 (29.6)	51% (27-74%)	39% (23-56%)	25% (0-55%)
33 (32.8)	58% (40-75%)	34% (23-45%)	26% (6-48%)
34 (34.3)	56% (38-73%)	34% (24-44%)	29% (11-47%)
35 (35.2)	54% (37-70%)	32% (22-42%)	26% (11-44%)
36 (35.9)	54% (36-70%)	33% (23-42%)	23% (9-38%)
37 (36.6)	55% (36-74%)	31% (21-41%)	21% (9-35%)
38 (38.4)	40% (19-62%)	28% (18-39%)	12% (2-22%)
39 (39.2)	35% (12-60%)	25% (15-37%)	8% (0-18%)
40-44 (41.4)	22% (0 to 67%)	14% (3 to 28%)	8% (0 to 20%)

7-year moving groups are utilized for ages 33-39 years. Ages 25-32 years and 40-44 years are grouped as fixed groups. Since linear interpolation is not used to center data on each age integer the data for ages 33 through 39 differ slightly from Figure 3 which does use linear interpolation.

### Validation Study

**Figure 2:**
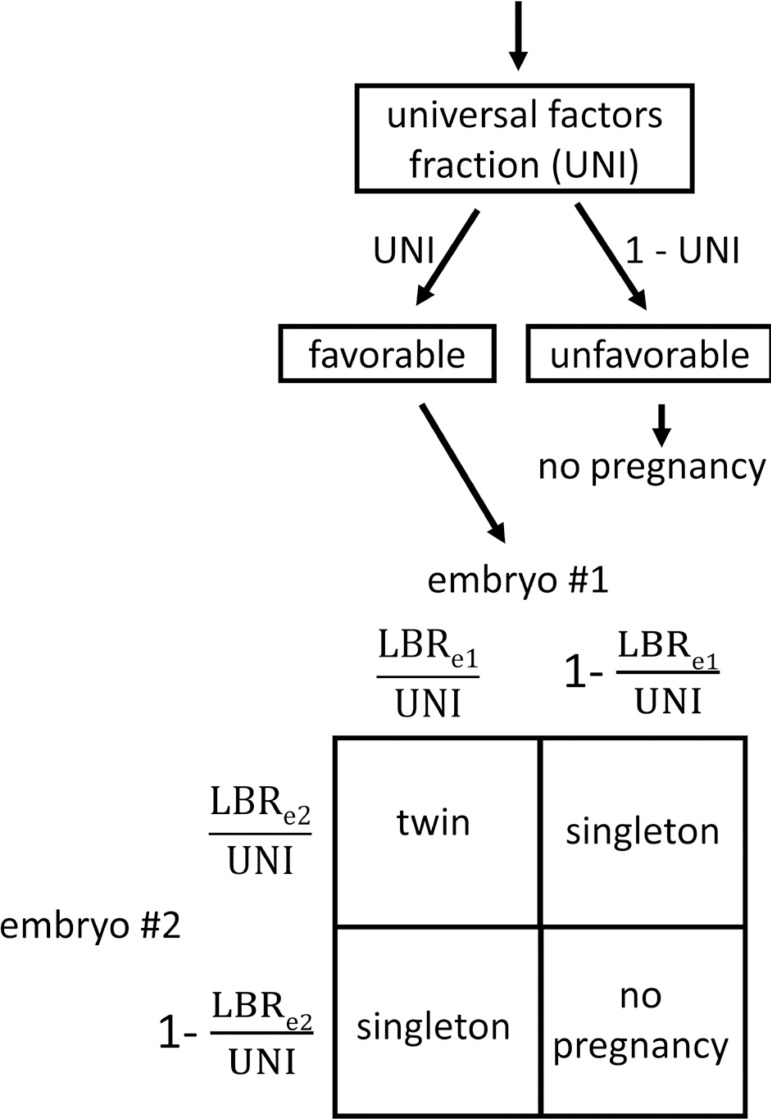
Logic for predicting outcomes of a double embryo transfer incorporating a universal factors fraction and live birth rates for embryo 1 and embryo 2. The universal factors fraction is a fraction from 0 (never favorable) to 1 (always favorable). The probability of each outcome is equal to the product of the terms next to the corresponding arrows above and the corresponding terms on the perimeter of the square. For example, the probability of twins is UNI×LBRe1UNI×LBRe2UNI. This same logic can be applied to transfer of more than two embryos. UNI = universal factors fraction; LBRe1 = live birth rate for embryo 1; LBR_e2_ = live birth rate for embryo 2.

**Figure 3:**
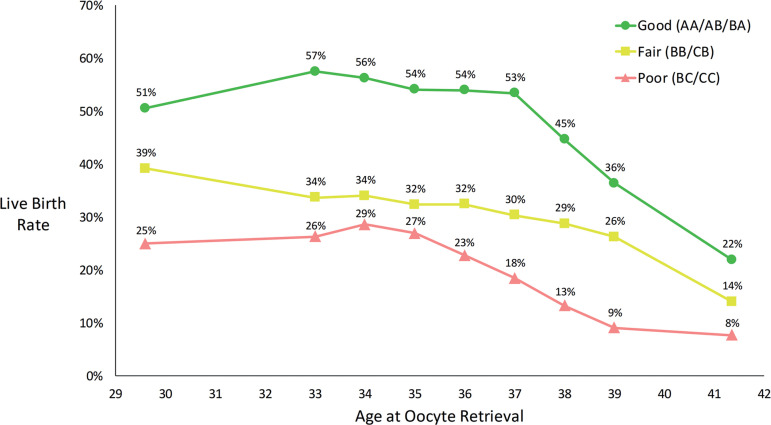
Live birth rates per blastocyst by age and overall morphology grade. 7-year moving groups are utilized for ages 33-39 years. Ages 25-32 years (average age 29.6) and 40-44 years (average age 41.4) are grouped as fixed groups. Linear interpolation is used to center live birth rates on the age of interest for ages 33-39 years.

We have previously validated an embryo transfer model that predicts rates of singleton and twin deliveries based on embryo live birth rates and universal factors that affect all embryos transferred concurrently. This model is defined by the logic in Figure 2. The previously validated model did not account for embryo morphology. For this reason, we performed an additional validation study based on the best fit live birth rates in Figure 3 and the logic in Figure 2 using computer simulation in MATLAB. We used 45 blastocyst transfers from May 2018 through December 2018 as the test data. This represents a test dataset that is entirely separate from the training dataset shown in Figure 1. The actual rates of singleton, twin, and total live birth were not significantly different from the predicted rate probability distributions (*p* = 0.37, *p* = 0.29, and *p* = 0.36, respectively). The MATLAB code for the validation study is accessible through the referenced Mendeley Data dataset (Awadalla, 2020).

## RESULTS

Live birth rates decreased with increasing age and worsening embryo morphology. The highest live birth rates for all transfers combined were seen for embryos with morphologies of AA/AB/BA (good) followed by BB/CB (fair) and the lowest live birth rates were seen with morphologies of BC/CC (poor). Although embryos with worse morphological grading were from older patients on average, this grouping of embryo morphology persisted when the analysis was restricted to patients less than 38 years old where the average age for each morphology was similar (Table 3). An overall morphology grade of good was assigned to 23% of embryos, fair to 53%, and poor to 24% (Table 2).

In the 25 to 32-year age group the average age was 29.6 years and the LBRs were 51%, 39%, and 25% for good, fair, and poor embryos respectively. In the 40 to 44-year age group the average age was 41.4 years and the LBRs were 22%, 14%, and 8% for good, fair, and poor embryos respectively. LBRs for each age and overall morphology grade using 7-year moving centered age groups for ages 33 through 39 years are shown in Figure 3. 95% confidence intervals for live birth rates for each age group and overall morphology grade are given in Table 5.

## DISCUSSION

Using a system of equations approach and solving with linear algebra allows for determination of live birth rates from both single and multiple embryo transfers. This approach was used to group embryos into good (AA/AB/BA), fair (BB/CB), and poor (BC/CC) overall morphology grade categories. The distinction between the fair and poor groups in this study favored the TE grade as a more important determinant of LBR than ICM grade. Most other studies have also found TE grade to be more important than ICM grade in predicting live birth (Ahlström *et al*., 2011; Bos-Mikich *et al*., 2016; Hill *et al*., 2013; Honnma *et al*., 2012; Thompson *et al*., 2013). One study found that TE grade and expansion stage were better predictors of live birth than ICM grade (Ahlström *et al*., 2013). On the other hand, a study by Subira *et al*. (2016) found that ICM grade was associated with LBR while TE grade was not. Another study found that expansion stage was more predictive of live birth than TE or ICM grade (Van Den Abbeel *et al*., 2013). Still another group chose to use blastocyst expansion in their model and exclude TE and ICM scores due to partial dependence on blastocyst expansion (Desai *et al*., 2016).

**Figure 4:**
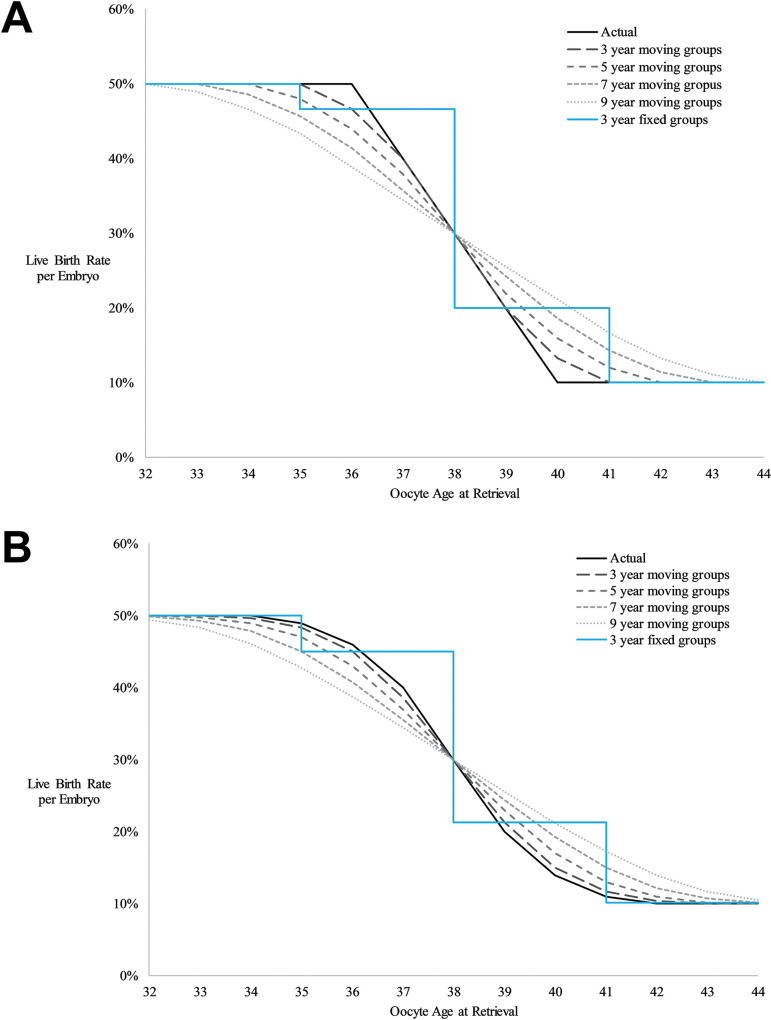
Demonstration with example data of the effect of 3-year fixed and 3, 5, 7, and 9-year moving groups on predicted live birth rates for (A) abrupt changes in live birth rate with age and (B) gradual changes in live birth rate with age.

Moving centered age groups allow for more precise determination of live birth rates for a specific age by including more data in each age group. For example, rather than using data from patients aged 35-37 years to determine live birth rates for a 37-year-old, an age group of 35-39 years is centered on 37 years and includes 5 years of data rather than only 3 years of data. In our model using fixed 3-year groups, a patient who undergoes transfer of a single good quality blastocyst would have a predicted live birth rate of 60% at age 37 years and a decrease to 35% at age 38 years (Table 4). With 7-year moving groups the same patient has a predicted live birth rate of 53% at age 37 years with a more realistic decrease to 45% at age 38 years (Figure 3). We evaluated 1, 3, 5, 7, and 9-year moving groups and chose 7-year groups as the best model because this balanced smoothing random variation in the data with a desire to use as small of an age range as possible. We performed a simple evaluation of the effect that different sizes of moving groups from 3 to 9 years would have on the best fit live birth rate and based on this analysis feel that 7-year moving groups are reasonable (Figure 4). We assume that with moving centered age groups the age range is narrow enough that inclusion of younger and older patients offset each other. With moving centered age groups, there is no assumption that the overall relationship between age and outcome is linear as is made with linear regression.

In addition to live birth rates per embryo, universal factors affecting all concurrently transferred embryos must be taken into account when determining the number of embryos to transfer. This concept was described as “the upper limit of the overall pregnancy rate if a large number of healthy embryos were transferred” by Speirs *et al*. (1983). Other terms used to describe this concept are “uterine receptivity” and “universal factors.” With this model, when universal factors are not favorable no embryos will implant. On the other hand, when universal factors are favorable, all embryos have an increased likelihood of implantation.

Speirs *et al*. (1983) estimated an upper limit of the overall pregnancy rate of about 70% for fresh embryo transfers, but the exact limit is not known with certainty. A “upper limit” or “uterine receptivity” rate of 70% would mean that in 30% of embryo transfers no implantations occur due to uterine factors or factors that affect all embryos. The remaining 70% of the time all embryos implant independently of other embryos but with increased implantation rates. For example, consider an embryo with an overall live birth rate of 50%. This embryo would have a 0% live birth rate in 3 out of 10 embryo transfers. In the remaining 7 out of 10 transfers the live birth rate is increased to 71.4% since 50/0.7 = 0.714. The overall live birth rate is still 50% when all 10 embryo transfers are considered as a group since 0 x 0.3 + 71.4 x 0.7 = 50. Modeling universal factors in this way does not affect outcome predictions for single embryo transfers but is needed to prevent systemic prediction errors for multiple embryo transfers (Torsky *et al*., 2005).

**Figure 5:**
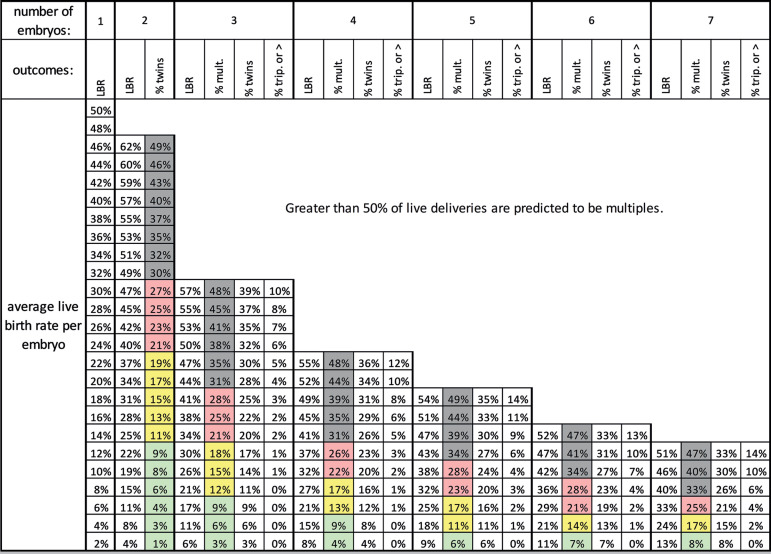
Predicted rates of live birth and multiple gestation after transfer of multiple embryos for a universal factors fraction of 0.70. The multiples column results are shaded green, yellow, red, or gray to indicate the risk of multiples at delivery (0-9%, 10-19%, 20-29%, or ≥ 30% respectively). LBR = total live birth rate per embryo transfer; % mult. = percentage of live deliveries that are multiples; % twins = percentage of live deliveries that are twin deliveries; % trip. or > = percentage of live deliveries that are triplets or greater.

Estimations from modeling our clinic data give an “upper limit” of 68% for fresh embryo transfers and 75% for frozen embryo transfers. To incorporate this into embryo transfer predictions we can use the logic shown in Figure 2 which denotes this upper limit by a universal factors fraction (UNI). Using a UNI value of 0.70 (the fractional representation of 70%) for both fresh and frozen embryo transfers simplifies the model. This logic can be represented in a table with rows for the average rate of live birth per embryo and columns for predicted outcomes based on the number of embryos transferred (Figure 5). Using the average rate of live birth per embryo rather than the individual rates of live birth is a reasonable and conservative simplification since this will slightly overestimate the risk of twins. This allows for use of Figures 3 and 5 together to quickly calculate a quantitative risk of multiple birth for a given blastocyst transfer.

### Limitations

This study is limited in that only age and embryo morphology were considered in an attempt to simplify the model. Transfer cycle type (fresh or frozen cycle) was not taken into account because our clinic blastocyst transfers have similar live birth rates for both cycle types. Additionally, our current dataset makes concurrent assessment of cycle type and embryo morphology challenging. We also did not take into account blastocyst expansion score (stage) but did exclude transfer of embryos with delayed blastulation on day 7. Evaluation of additional factors such as these could be performed using a larger dataset. Just as it is uncertain how applicable aggregate data from multicenter databases are to individual clinics, it is also uncertain how applicable data from one clinic is to embryo transfers at other clinics.

## CONCLUSIONS

A system of equations, or linear algebra, approach allows for concurrent analysis of both single and multiple embryo transfers. For a given age group size such as 7 years, moving centered age groups provide better resolution of age-related changes in live birth rates than fixed age groups. Use of moving centered age groups may be preferable to linear regression as there is no assumption of an overall linear relationship between age and outcomes. This model allows for blastocyst morphology to be taken into account to quantitatively estimate the risk of multiples for a planned embryo transfer.

## Supplemental Methods

### MATLAB Programs

For reproducibility, the MATLAB code used to generate all of the pertinent data for figures, tables, and the validation study is being provided with this manuscript through Mendeley Data (Awadalla, 2020). All of the data analysis can be performed by running the file “generate_data.m” with MATLAB. Descriptions for each of the 5 included MATLAB programs are included in the comments section at the beginning of each program.

### Methodology for Determination of Confidence Intervals for Live Birth Rates

Bootstrapping was used to simulate embryo transfers based on the logic in Figure 2 to determine 95% confidence intervals (CIs) for the live birth rates. A random number generator was used to simulate universal factors that affect all embryos such that if universal factors were not favorable no embryos implanted. If factors were favorable, embryos were assumed to implant independently with a live birth rate of $\frac{\mathrm{LBR}}{\mathrm{UNI}}$ where UNI denotes a universal factors fraction and LBR denotes the overall live birth rate of an embryo. A universal factors fraction of 0.70 was used to model favorable universal factors 70% of the time. This is the approximate value for our embryo transfers based on internal data and that of others (Speirs *et al.*, 1983). If the uterine factors were favorable, random number generation was again used to determine if each embryo would implant or not based on the logic in Figure 2 which is an example of logic for a double embryo transfer. For each group of embryo transfers the outcomes were simulated 100,000 times and for each simulation best fit live birth rates were calculated to create a probability distribution of predicted live birth rates. 95% CIs for good, fair, and poor embryo quality live birth rates were taken from the probability distribution determined by the simulation. If the lower bound 95% CI limit was less than 0 this was reported as 0% since negative values are not biologically plausible and likely reflect low numbers of embryos in certain groupings. The MATLAB code for the computer simulation is being provided through Mendeley Data and the analysis can be performed by running the “generate_data.m” file (Awadalla, 2020). The 95% CIs for live birth rates are given in Table 5 for each age group.

### *In Vitro* Fertilization Protocols

Our protocol for ovarian stimulation, oocyte collection, IVF, ICSI, embryo culture, and fresh embryo transfer has been described previously (Awadalla *et al*., 2020).

### Vitrification Technique

Blastocysts of grade 1CC or higher were considered for vitrification. Blastocyst vitrification was performed according to the I.C.E. Blastocyst Vitrification Protocol (Innovative Cryo Enterprises LLC, Linden, NJ). The Cryotop vitrification device (KitaZato, Tokyo, Japan) was used for blastocyst vitrification. Prior to vitrification, blastocysts were collapsed with laser pulses directed at a thin portion of the trophectoderm away from the inner cell mass to prevent ice crystal formation. Vitrification was performed 10-30 minutes after the embryo was collapsed.

### Frozen Embryo Transfer

The majority of frozen embryo transfers occurred in programmed cycles. Starting on day 2 of the menses a baseline transvaginal ultrasound (TVUS) was performed and 2mg oral estradiol was administered twice a day for 6 days then three times a day until embryo transfer. On approximately day 11 of estradiol a serum progesterone and endometrial thickness by TVUS were measured. Cycles were cancelled if the progesterone was greater than or equal to 1.5 ng/mL. Additional days of estradiol were prescribed if the endometrial thickness was less than 7.0mm. If the serum progesterone was less than 1.5 ng/mL and the endometrial thickness was equal to or greater than 7.0mm, then IM and vaginal progesterone was started. IM progesterone was started with 50mg IM progesterone in ethyl oleate starting at 9PM (day 1 of progesterone). Subsequent days of progesterone consisted of IM progesterone at 9AM and 200mg micronized progesterone vaginally at 1PM and 9PM. Blastocyst transfers were scheduled on day 6 of progesterone (approximately 108 hours after the first IM progesterone dose). This timing was adjusted to the implantation window if a patient had previously had an endometrial biopsy for receptivity testing. Doxycycline 100mg orally was prescribed twice a day starting in the morning the day prior to the embryo transfer for a total of three doses. After embryo transfer, patients were prescribed oral estradiol 2mg twice a day, 50mg IM progesterone in ethyl oleate once daily, and 200mg micronized progesterone vaginally twice a day for 13 weeks. The first serum HCG was measured 9 days after blastocyst transfers.
